# Enhanced sampling of protein conformational states for dynamic cross‐docking within the protein‐protein docking server SwarmDock

**DOI:** 10.1002/prot.25851

**Published:** 2019-11-20

**Authors:** Mieczyslaw Torchala, Tereza Gerguri, Raphael A. G. Chaleil, Patrick Gordon, Francis Russell, Miriam Keshani, Paul A. Bates

**Affiliations:** ^1^ Biomolecular Modelling Laboratory The Francis Crick Institute London UK; ^2^ Hadean Supercomputing Ltd London UK

**Keywords:** Aether Engine, CAPRI, conformational selection, conformational states space sampling, cross‐docking, DFIRE2, induced fit, normal modes, protein‐protein docking, protein‐protein interactions, SwarmDock

## Abstract

The formation of specific protein‐protein interactions is often a key to a protein's function. During complex formation, each protein component will undergo a change in the conformational state, for some these changes are relatively small and reside primarily at the sidechain level; however, others may display notable backbone adjustments. One of the classic problems in the protein‐docking field is to be able to a priori predict the extent of such conformational changes. In this work, we investigated three protocols to find the most suitable input structure conformations for cross‐docking, including a robust sampling approach in normal mode space. Counterintuitively, knowledge of the theoretically best combination of normal modes for unbound‐bound transitions does not always lead to the best results. We used a novel spatial partitioning library, Aether Engine (see Supplementary Materials), to efficiently search the conformational states of 56 receptor/ligand pairs, including a recent CAPRI target, in a systematic manner and selected diverse conformations as input to our automated docking server, SwarmDock, a server that allows moderate conformational adjustments during the docking process. In essence, here we present a dynamic cross‐docking protocol, which when benchmarked against the simpler approach of just docking the unbound components shows a 10% uplift in the quality of the top docking pose.

## 1 INTRODUCTION

Specific protein‐protein interactions are both ubiquitous and essential within biological processes, ranging from immune system surveillance to tissue development and repair. Invariably, changes in the conformational state of the components of protein complexes do occur upon complex formation.^1^, ^2^ However, it is difficult to a priori predict the extent of such changes. Nevertheless, much research has provided guiding principles, the most notable for which is the concept of conformational selection and induced fit.^3^ Conformational selection can be defined as a set of states, with likely varying degrees of stability, and therefore occupancy, which a protein may sample before complexation with a binding partner. Induced fit, on the other hand, can be defined as the influence that one binding partner may have on the other, often in the final stages of docking, where conformational states may be stabilized that are not possible for the free unbound components to sample. Although recent work has uncovered some interesting trends,^4^ just how important the above two mechanisms are for the complex formation of any one particular protein receptor/ligand pair remains difficult to estimate. In the context of fully automated docking servers, which often cannot capture the full extent of conformational changes displayed by unbound components, it would be desirable to be able to precalculate unbound conformations that are more predisposed toward their bound conformational states, and subsequently take such predictions into account within the automated docking protocol. One relatively successful protocol, in which the above has been partially implemented, is that of cross‐docking. A number of cross‐docking methodologies have been reported^5^, ^6^, ^7^; however, once a number of diverse receptor and ligand conformations have been selected, the docking process is typically rigid body. Here, we ask the question of how well might flexible cross‐docking perform: that is, selection of viable alternative conformations for docking, and then employment of a docking methodology that enables limited flexibility in docking the selected conformations, here termed as “dynamic cross‐docking.” Clearly, many thousands of preselected conformations could potentially be cross‐docked; however, most automated docking algorithms, with some degree of dynamic flexibility allowed during the docking process, would struggle to search such a large space within a turnaround time suitable to run as a publicly available server.

Moreover, sampling of the conformational states space is a tedious process as it depends on the single potential calculation time and the number of samples. With increasing numbers of normal modes, sampling boundaries, and granularity of sampling, the search task rapidly becomes intractable when utilizing standard high‐performance computer (HPC) hardware, thereby demanding multicore parallelization and advanced knowledge in distributed computing.

As the importance of the given nontrivial normal mode is generally unknown when the bound structure cannot be compared with unbound, we decided to start with a naïve protocol—docking the conformations at the extreme magnitudes (in positive and negative directions) along the first nontrivial normal mode. This required nine docking runs per complex. After that, we investigated whether knowledge of the theoretically best combination of normal modes improves the docking results. Finally, we employed a method for sophisticated inputs generation, which involves sampling and analyzing the multidimensional normal modes space; here restricted to the first three nontrivial normal modes, but the approach is extendable to any number of dimensions. This enhanced approach requires only four docking runs per complex, but includes a generally expensive sampling step. Therefore, we employed a novel spatial partitioning library to efficiently search the protein conformational states space to reduce time needed to produce a minimal set of conformations for cross‐docking. All protocols are compared on a set of 55 unbound receptor/ligand pairs, and one recent, difficult CAPRI target, for which the coordinates of the complex are available.

## 2 MATERIALS AND METHODS

### 2.1 Overview

The methodology presented here is aimed at identifying a plausible route to identifying the best conformation for each of a receptor and ligand pair to input to a docking server to enhance the quality of resulting docked poses. Our own protein‐protein docking server, SwarmDock,^8^ a server that allows moderate levels of conformational change, is used to cross‐dock selected receptor and ligand conformations. Conformations for docking are selected according to three protocols, as outlined later.

### 2.2 The data set

The 55 complexes from the docking benchmark version 5,^9^ for which there are both experimentally determined unbound receptor and ligand components, as well as the experimentally determined complex, were used in this analysis. In addition, one difficult CAPRI target, T131 (6GBG), was also selected to test the methodology: a CAPRI case for which the SwarmDock server previously failed to return a correct docked pose. Prior to the methods described after the original Benchmark 5 was manually curated to repair missing atoms and residues, these data accompany the DYNACROSS decoy set for scorers and we have generated as a part of this work (see Supporting Information for details).

### 2.3 Calculation of elastic normal modes

To investigate the potential range of conformational states, a receptor/ligand pair may sample upon forming a complex we calculate from their unbound states as determined experimentally and their individual set of normal modes. Representing protein conformations as a combination of normal modes is a well‐established methodology for both understanding protein‐fold conformational state transitions and employing in protein docking pipelines.^10^, ^11^, ^12^, ^13^, ^14^ More specifically, a full‐atom elastic network normal mode calculation was performed on each unbound receptor/ligand pair with the program elNemo.^15^ Throughout this work, references to the lowest modes indicate the lowest nontrivial modes: the first six trivial modes, with zero frequency that corresponds to rigid‐body rotation and translation, are not considered.

### 2.4 Calculation of the solvent‐accessible surface area

To investigate how docked pose quality may have improved when normal modes were applied to the inputs, we calculated the solvent‐accessible surface area (SASA) using the FreeSASA^16^ Python package (https://freesasa.github.io/).

### 2.5 Dynamic cross‐docking with SwarmDock

All receptor/ligand pair conformations selected for cross‐docking, see later for selection protocols, were submitted to the SwarmDock server.^8^ The server and underlying algorithm have been described previously.^8^, ^17^, ^18^ Briefly, the server models any missing residues and atoms, reformats nonstandard residues, and minimizes the starting conformation. The core algorithm can be described as a modified version of the particle swarm optimization (PSO) algorithm,^19^ which is employed to optimize the binding energy, as determined using the DComplex potential.^20^ Each particle search vector consists of the position and orientation of the ligand, as well as normal mode coefficients, corresponding to the five lowest frequency nontrivial modes, to model the conformation of the receptor and the ligand, calculated by the elNemo program.^15^ After each PSO iteration, the lowest energy member undergoes a local optimization.^21^ Starting swarms are released from approximately 120 evenly spaced positions around the receptor, and swarms are run four times from each of these positions.

It is necessary to mention that the quality of docked poses generated by SwarmDock Server may sometimes be of lower quality than other leading methodologies, for example, ClusPro.^22^ However, here we aim to evaluate “dynamic cross‐docking,” a general methodology that can be applied to any other docking approaches and thus support the wider docking community.

### 2.6 Selection protocols

#### 2.6.1 P1: Naïve approach: extremes of the conformation space of each unbound receptor/ligand pair

Most of the conformational space between unbound and bound conformations can be accounted for within the first few lowest mode combinations of the unbound.^18^ First, for each unbound set of coordinates, the maximum extent along each of the three modes separately (maximum mode amplitude) is calculated, in both negative and positive directions. Preliminary runs of this protocol indicated some unwinding of secondary structure elements, and while this is tolerable to an extent, indeed may naturally occur upon protein complexation, a high degree of such unwinding would clearly destabilize the binding partners. Therefore, it was deemed prudent, for both this protocol and protocol P3 described later, to monitor the amount of unwinding and set a threshold for its occurrence. Hence, each extended conformation is minimized via the program CHARMM^23^ and the secondary structure conservation (see later) calculated by comparing the initial and new conformation secondary structure fingerprints. Preliminary runs also suggested initial maximum mode amplitudes of 100 and −100 in the positive and negative directions, respectively, and provided a good balance between the energy of an expanded conformation and extent of secondary structure unwinding. If the structure is minimized within a secondary structure threshold, the maximum mode amplitude is doubled, otherwise, it is halved. The process is complete when the minimum and maximum amplitude ranges match. As discussed earlier, the secondary structure conservation is an additional constraint to ensure that protein folds do not notably unwind; the percentage of secondary structure for the new coordinates is checked to ensure that the percentage of secondary structure relative to the original unbound coordinates does not deviate by more than 10% for any of the three main secondary structure states, helix (H), beta strand (E), and coil (C), as calculated by the program DSSP^24^ (all secondary states other than H and E were converted to C) and called via Biopython (https://biopython.org/).

In this protocol, only the lowest frequency mode for each receptor and ligand is searched, and the maximum extent (mode amplitude) is determined given the constraints described earlier. The first of the nine runs consists of a normal SwarmDock run of docking the unbound coordinates of receptor (Ru) and ligand (Lu). The 9 cross‐docking runs are performed as follows: (Ru) with ligand maximum extension of the lowest mode in the positive direction (L+); Ru with maximum extension in the negative direction (L−); similarly, for (Lu), cross‐docking is performed with the receptor conformation extended to a maximum in the positive direction (R+) and the negative direction (R−); therefore, the nine cross‐docking runs were as follows: (Ru):(Lu), (Ru):(L+), (Ru):(L−), (R+):(Lu), (R+):(L+), (R+):(L−), (R−):(Lu), (R−):(L+), and (R‐):(L‐).

(Ru):(Lu) is additionally reported as P0, which shows the results of running a basic docking from unbound inputs comparing with cross‐docking approaches.

#### 2.6.2 P2: Theoretically best normal modes combination as input

The vector of coefficients, *β*, that represent the closest fit between unbound and bound receptor or ligand conformational states, can be formulated in matrix notation as follows:

(1) $$\beta ={\left(M^{T}M\;\right)}^{-1}\;M^{T}T,$$

where *M* is an *m* × *n* matrix of *m* normal modes and *n* coordinates (unbound), and *T* is a vector of the difference in coordinates, after superposition, between bound and unbound conformations. For the full mathematical derivation, please see Moal et al.^18^

As a benchmark, the theoretically best combination of the three lowest frequency modes for both receptor and ligand, see Table 1, is used to construct the input conformations to the server. For this protocol, there is no need to monitor unwinding of secondary structure elements as we are simply interested in the parsimonious route between unbound and bound conformations and the combination of mode amplitudes to achieve this. It should be noted that the SwarmDock server is not using any information on known bound structures. The above procedure is for benchmarking purposes only and to investigate whether knowledge of the best combination can be helpful in docking, which is of interest for development of in silico predictors from unbound structures.

**Table 1:** The 55 targets from Benchmark 5^9^ and the CAPRI target T131, PDB code 6GBG^32^ (in bold font), used in this analysis

PDB complex	Type	IRMSD	Difficulty	Receptor			Ligand			Protocols			
				M1	M2	M3	M1	M2	M3	P0	P1	P2	P3
3EOA	A	0.39	RB	252.64	26.56	57.65	7.52	−2.17	10.17	A	A	A	A
3BIW	OX	0.39	RB	−16.38	0.35	25.38	5.04	1.70	4.62	A	A	A	**M**
4 M76	OR	0.43	RB	−10.61	−4.04	1.14	−3.90	−7.68	−3.54	I	**A**	I	I
1JTD	EI	0.44	RB	1.03	4.13	−3.65	0.93	3.08	3.83	I	**M**	**A**	**A**
3L5W	A	0.48	RB	−44.49	6.84	12.02	−7.57	−11.72	−29.54	I	**A**	I	**A**
3MXW	A	0.48	RB	50.73	−18.51	1.23	3.48	−1.58	−2.00	M	M	M	M
4G6M	A	0.49	RB	−13.69	9.58	−2.87	−1.53	−0.65	6.12	M	M	M	M
3RVW	A	0.50	RB	−8.70	−11.37	−3.34	5.29	10.70	6.83	I	I	I	A
3PC8	ER	0.50	RB	−0.14	2.99	−1.36	−13.63	−9.09	−0.55	H	H	H	H
3VLB	EI	0.51	RB	−8.10	13.12	−22.29	4.21	−10.68	−2.61	M	M	M	M
3P57	OX	0.53	RB	−2.48	−0.09	−0.67	19.82	16.01	21.28	A	A	A	A
2GTP	OG	0.54	RB	−4.65	−6.69	−21.10	−4.22	−3.30	−5.16	H	H	**M**	H
2YVJ	ER	0.60	RB	4.32	10.89	−10.91	−0.42	3.78	1.52	M	M	M	M
4G6J	A	0.61	RB	−166.10	12.60	−21.70	−3.83	0,76	−0.60	M	M	M	M
1EXB	OX	0.62	RB	−4.45	12.67	−13.45	23.87	4.53	10.04	H	H	H	H
3 K75	ER	0.64	RB	−28.65	−0.84	−13.82	0.16	−1.49	−2.98	M	M	M	M
4H03	ES	0.68	RB	−20.74	−11.31	−12.49	12.85	42.78	38.05	I	**A**	I	I
2GAF	ER	0.69	RB	−2.24	−4.38	22.32	3.03	12.19	−5.00	M	M	M	M
3A4S	EI	0.72	RB	24.13	6.77	1.90	−7.93	15.26	−6.36	M	M	M	M
3HMX	A	0.73	RB	−176.10	47.67	6.96	30.28	36.90	−4.80	A	A	A	A
BP57	OX	0.74	RB	1.63	1.21	0.47	20.26	15.67	20.74	I	I	I	I
4GXU	A	0.78	RB	27.41	6.96	−3.60	171.39	28.07	24.10	I	**M**	**A**	**A**
3H2V	OX	0.80	RB	12.89	0.25	−4.22	9.83	−1.60	−5.86	I	**A**	I	**A**
4DN4	A	0.81	RB	−177.76	−44.97	−0.31	3.66	3.74	1.38	M	M	M	M
3LVK	ER	0.81	RB	−23.09	−3.05	9.71	−7.67	2.69	−3.22	M	M	M	M
4HX3	EI	0.90	RB	21.85	6.44	−15.03	3.11	4.15	−1.40	M	M	M	M
CP57	OX	0.91	RB	−4.17	5.42	−1.18	−0.17	0.01	0.05	M	M	M	M
4FQI	A	1.08	RB	142.03	−39.70	26.35	−118.89	−15.29	9.26	A	A	A	A
2W9E	A	1.13	RB	−21.10	7.69	−16.40	6.19	11.00	0.59	I	**A**	I	I
1 M27	OX	1.22	RB	2.28	−6.43	1.43	2.70	−4.82	4.89	A	**M**	A	**M**
2VXT	A	1.33	RB	−58.55	−36.44	28.40	−2.95	−9.40	4.96	I	**A**	I	**A**
2X9A	OR	1.33	RB	1.94	−0.75	−10.04	−0,71	−1.19	−2.42	A	A	A	A
2A1A	ES	1.35	RB	0.05	0.08	0.96	−15.94	−11.30	5.92	A	A	A	A
3EO1	A	1.37	M	80.74	−25.14	−11.74	53.36	9.39	8.74	A	A	A	A
3DAW	OX	1.49	M	50.29	−1.68	23.33	20.86	−4.81	5.40	A	A	A	A
4IZ7	EI	1.56	M	12.24	27.11	20.68	11.60	13.81	−8.48	I	I	I	I
4LW4	ES	1.60	M	−19.77	3.31	−9.73	−39.72	−8.93	39.82	A	A	A	A
4JCV	OX	1.62	M	225.96	22.95	−11.33	25.79	−21.10	−34.79	A	A	**I**	**M**
3BX7	OX	1.63	M	1.55	22.06	7.36	−4.11	−12.48	−4.21	A	A	A	A
3HI6	A	1.65	M	−140.53	−41.11	−7.63	16.36	−15.11	−18.27	I	I	I	I
3S9D	OR	1.69	M	1.17	−0.40	48.63	16.33	1.81	−4.70	M	M	M	M
3AAA	OX	1.78	M	−77.49	58.28	32.22	2.94	−6.71	0.20	A	A	**I**	A
3V6Z	A	1.83	M	−21.01	16.58	6.74	−56.12	0.19	50.15	I	I	I	I
3G6D	A	1.86	M	361.98	6.16	−18.48	9.88	−33.32	−7.28	A	A	**I**	**M**
3R9A	OR	1.91	M	−0.45	−2.97	−7.86	70.43	−41.64	23.27	A	A	A	A
BAAD	OX	2.00	M	−11.45	−56.21	12.49	4.37	1.08	−0.70	I	I	**A**	I
4FZA	ER	2.04	M	13.22	30.36	4.74	−0.40	8.56	11.01	A	A	**I**	A
3SZK	OX	2.10	M	9.49	12.85	−24.44	27.75	−15.85	−7.96	M	M	**I**	M
3 L89	OR	2.51	D	−8.77	−10.06	−0.11	−121.96	−4.27	−55.80	A	A	**I**	A
3F1P	OX	2.52	D	−0.97	−5.62	−3.15	3.48	4.08	4.59	M	M	M	M
3FN1	ER	3.65	D	4.29	−0.82	0.58	33.71	7.24	17.34	A	**M**	**M**	A
3H11	ER	3.79	D	32.62	−12.08	−23.19	−11.01	6.84	−5.52	I	I	I	I
1RKE	OX	4.25	D	−12.68	−11.57	−81.11	−52.27	−6.60	−8.65	A	A	**I**	A
3AAD	OX	4.37	D	15.80	−58.67	6.39	−8.57	−6.27	3.50	I	**A**	**A**	**A**
4GAM	ER	5.79	D	15.65	25.17	−3.58	8.57	−67.77	43.11	I	I	I	I
**6GBG**	OX	1.82	M	−21.65	4.64	24.68	−5.52	−11.90	−3.95	I	**A**	I	**A**

*Notes: Type*: protein function categories as described in Vreven et al.^9^: A, antibody‐antigen; EI, enzyme‐inhibitor; ES, enzyme‐substrate; ER, enzyme complex with a regulatory or accessory chain; OG, others, G‐protein containing; OR, others, receptor containing; OX, others, miscellaneous. IRMSD: root‐mean‐squared deviation of Cα atoms of residues at the receptor/ligand interface, calculated after finding the best superposition of bound and unbound. *Difficulty*: three categories for expected docking difficulty as defined by IRMSD; rigid‐body (RB), IRMSD ≤ 1.35 Å; medium (M), IRMSD > 1.35 Å and IRMSD ≤ 2.5 Å; and difficult (D), IRMSD >2.5 Å. Next, amplitudes of the three lowest mode for the receptor, followed by the amplitudes of the first three lowest modes of the ligand, as calculated by Equation (1); values highlighted in bold if the first amplitude for a receptor or ligand is higher than the following two. Finally, columns P0‐P3 show the best obtained docked pose quality (incorrect, acceptable, medium, or high) for basic docking runs (from unbound; (Ru):(Lu) run from P1), naïve approach (P1), theoretical combination of normal modes input approach (P2), and sophisticated sampling approach (P3). When comparing with protocol P0, transitions with improving quality are highlighted in blue, and the ones worsening the quality are highlighted in red.

### 2.7 P3: Sophisticated sampling and selection of inputs

Here, we concentrate our efforts on mapping the conformational space explored by the first three lowest mode frequencies of each receptor, and each ligand, for every complex in our set of 56 targets. Once the maximum envelope has been calculated, separately for every normal mode, as described earlier, its volume was sampled at regular intervals along each axis by calculating the relative stability of each volume point, a point that represents the linear combination of the three modes. Sampling was performed with the distance‐dependent empirical atom‐atom potential program DFIRE2,^25^ a pair potential program that can rapidly assign the relative energy of a conformation.

In order to allow for energetic space comparison, the DFIRE2 potential values were normalized and multiplied by 10.0, so the normalized potential values are in the range between 0.0 for the lowest and 10.0 for the highest. This can be incorporated into a PDB‐like format using normal mode magnitudes as coordinates and normalized potential values as B‐factors. In this manner, the conformational state space can be easily visualized with up to three normal modes in the visualization graphics program VMD,^26^ using a color index as the fourth dimension (Figure 3).

After several trials with various thresholds, we isolated a subset of samples, where a cut‐off threshold for the normalized DFIRE2 energy score was set to <=5.0. The sample point at maximum distance in this normal mode space from the origin was then selected such that the sample point still maintains 90% of secondary structure conservation. When compared to protocol P0, this protocol leads to 4‐fold cross‐docking runs per complex: (R5):(L5), (Ru):(L5), (R5):(Lu), and (Ru):(Lu). The run results (Ru):(Lu) are already known from protocol P1 (and labeled as P0), so here we performed three docking runs and reused (Ru):(Lu) docking results for consistency.

It should be noted that even for just three modes, the sampled conformational envelope for each receptor and ligand pair is large. We selected a single conformation from each envelope as described earlier. Of course, many other sample points within these volumes could have been selected. In the context of the current work, it must remain a research aim to investigate the potential of selecting alternative conformations, perhaps with the aid of a feedback machine learning protocol that enabled capture of the finer energetic architecture of these volumes where subtle interplays between mode combinations along the docking trajectory could be revealed. It was initially hoped that eigenvalues, calculated along with the eigenvectors describing each mode,^15^ could be utilized to direct and scale each search for optimal configurations. However, a scatter plot of such eigenvalues against the theoretical amplitudes (obtained from P2, see Table 1) indicate, see Figure S1, that although there is a general trend for the smaller eigenvalues to correspond to larger mode amplitudes, a particular eigenvalue can map to a wide range of mode amplitudes, especially at the lower end of the eigenvalue range, thereby indicating that any guidance gleamed from eigenvalue ratios could only be considered weak.

### 2.8 Sampling efficiency benchmark

The DFIRE2 single potential calculation time is dependent on the number of atoms and is of approximately O(N^2^) complexity due to symmetry in interactions: our complexity analysis on the set of 110 proteins (55 complexes) of various sizes from Benchmark 5 gave O(N^1.98^). Additionally, the process scales linearly with the application of normal modes to change the atom coordinates. Moreover, sampling requires a huge number of calls to the potential calculation function, in this case dependent on the number of normal modes in use, granularity of sampling (step size), and the ranges for positive and negative extremes for every normal mode. As this would be a serious bottleneck for bigger proteins and normal mode spaces, we decided to use a novel spatial partitioning library to reduce the complexity of the calculation and accelerate processing time. Aether Engine's distributed octree is used here to decompose the atom space and speed‐up potential calculations; both on a single core and scaled across multiple processors (see Supporting Information for details).

We benchmarked this novel technology for proteins of various atom sizes. We prepared both (a) pure C++ single core and (b) Aether Engine based C++ codes and validated the correctness of implementation on around 38 000 of PDB structures generated during sampling. We first calculated the single potential calculation time (averaged over 100 runs) of the pure C++ implementation to estimate the number of samples to be computed in approximately 10 minutes. Then, after setting the ranges within three normal modes accordingly, we obtained the number of samples we had estimated were needed. We performed the benchmark both on a personal laptop and on Azure cloud VM, and this did not require any change to the code. As the results are comparable, here we present only cloud‐based results as the cloud resources allowed us to do 10 runs per type of calculation to generate the proper statistics; this is required because CPU performance is no longer fixed in contemporary machines (can be adjusted by the operating system depending on various factors) and as such out of the user's control. On pure C++ implementation, the calculation scales linearly with a number of samples. We then ran the same calculation on Aether Engine with various numbers of cores, using the VM equipped with Intel(R) Xeon(R) CPU E5‐2673 v4 @ 2.30GHz (64 logical cores, 2 sockets of 16 cores with two threads per core). The C++ code was compiled in release mode (contrary to the default cmake debug mode). “sched_setaffinity” was used to pin the processes to specific cores. The results are presented in Table S1.

A common property used to measure distributed systems is “speed‐up” *S*_*N*_, defined as the time to calculate on 1 CPU/core (*T*_*1*_) vs the time to calculate on *N* CPUs/cores (*T*_*N*_) (Equation 2):

(2) $$S_{N}=\frac{T_{1}}{T_{N}}.$$

The speedup of 1.0 means no change in calculation times, whereas the speedup below 1.0 suggests that there is no gain in using parallel processing, for example, due to overheads. Similarly, we can compare the time to compute off‐ and on‐Aether on 1 CPU/core. Traditionally, the theoretical limit for the speedup on *N* cores was *N* (eg, 4.0 for 4 cores). However, various special techniques, both hardware‐based like Hyper‐Threading, and software based, like spatial partitioning, are capable of boosting speedup even higher.

Using Aether Engine on a single core, a maximum 4.37 speed up in sampling has been achieved; this was beneficial for proteins above 4000 atoms, as well as increased acceleration because of the engine's ability to add parallelism to previously unparalleled problems (like DFIRE2 here) and scale to any number of CPUs/cores/machines without any change to the code. The speed‐ups on one core were between 4.37 for the protein containing 17 445 atoms and 0.57 for the protein containing 3012 atoms; respectively, on two cores, the numbers were 7.70 and 0.85, and on four cores, the numbers were 11.56 and 1.14. With the understanding that perfect scaling is unachievable in reality, these speed‐up analyses show the lower boundary of Aether outperforming pure C++ code implementation and that of the speed‐ups, thanks to spatial partitioning increase with the size of protein. This places Aether Engine as a tool for efficient analyses of bigger systems, while small systems (below 4000 atoms) can be still efficiently sampled with standard coding approaches.

These performance improvements, combined with the ability to efficiently use all resources available, mean that incorporating a spatial partitioning library, like Aether Engine, for sophisticated sampling in a publicly available server protocol would therefore negate any additional burden brought on by this preprocessing step. When combined with the reduction in number of cross‐docking runs (from 9‐fold to 4‐fold), P3 makes an interesting candidate methodology for future investigations.

### 2.9 Assessment of docking poses

For all of the abovementioned SwarmDock runs, the CAPRI assessment criteria^27^, ^28^ were used to assign quality to each docked pose. Three metrics were calculated, such as ligand root mean square deviation (LRMSD), interface root mean square deviation (IRMSD), and fraction of native contacts (FNAT). These metrics were then employed, using the same cut‐off values described in Mendez et al,^27^, ^28^ to classify each pose into one of four categories: high accuracy (H), medium accuracy (M), acceptable accuracy (A), and incorrect (I).

## 3 RESULTS AND DISCUSION

The 55 complexes from docking benchmark version 5,^9^ for which there are both experimentally determined unbound receptor and ligand components as well as the experimentally determined complex, were used in this analysis (see Table 1). Interestingly, as part of reporting this benchmark, four automated protein‐protein docking servers were benchmarked as to their success in finding solutions, three ab initio servers SwarmDock,^8^ pyDock^29^ and ZDOCK,^30^ plus one server for which biological constraints are typically used, HADDOCK^31^ (see Figure 1A in Vreven et al^9^). Disappointingly, for almost a quarter of the targets, and irrespective of the extent of conformational changes observed upon complex formation, all four docking servers were unable to return at least an acceptable docking pose within the top 50 ranked solutions, and for 8 of the targets an acceptable pose could not be found in the top 100 solutions. Although the abovementioned is certainly not an exhaustive list of publicly available docking servers, one notable admission being the well‐established and high‐performing ClusPro^22^ server, the following question can be asked of server performance: are servers generating any acceptable solutions for these supposedly more difficult cases, even if some are formally classified as rigid body? Therefore, in the context of one of the servers, SwarmDock, which is entirely within our own hands and can be experimented on with ease, we investigate the above question, and moreover, design a protocol to enhance the list of docked poses with potentially acceptable solutions (“acceptable”—as defined by the CAPRI assessment criteria, see the Materials and Methods section). For all protocols, we recorded both the highest FNAT docking pose and poses of the highest quality, as defined by the CAPRI scoring scheme.

**Figure 1:**
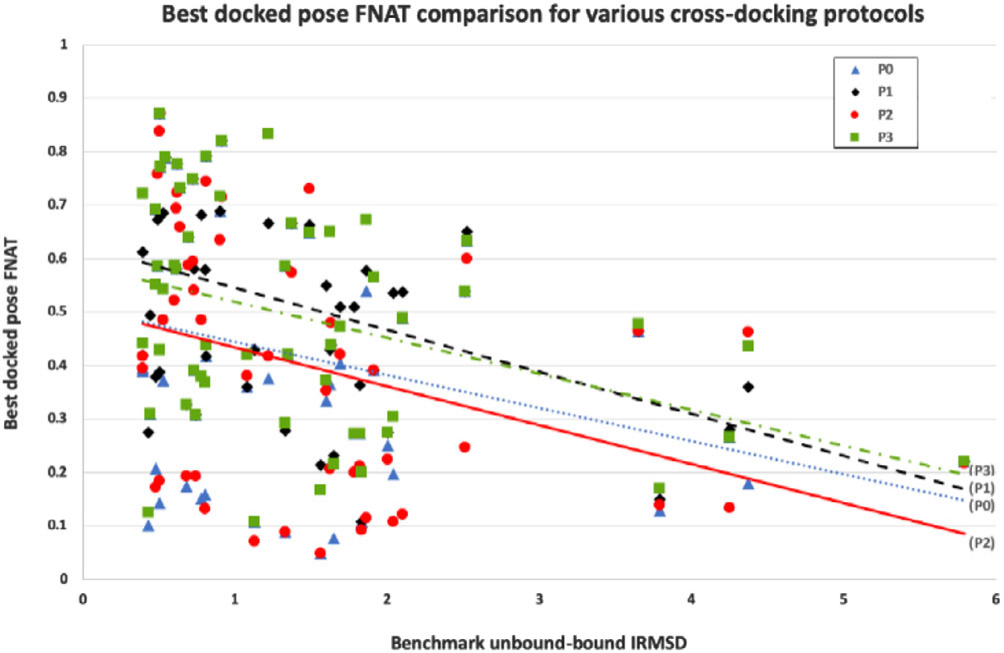
Plot of the best percentage of native contacts vs unbound‐bound IRMSD for the 56 Target when various protocols, from P0 to P3, are applied

Although we have entirely focused on designing protocols to better generate conformations that enrich the list of docked poses with higher quality models, rather than improve their final ranking, a preliminary analysis to determine whether we can identify the improved poses did not indicate significant improvements (data not shown).

This underlines that development of good ranking schemes continues to remain a major obstacle for server developers. Nevertheless, if a list of solutions does not contain high‐quality docking poses, no form of improved ranking will be able to enhance the overall docking pipeline, so we are encouraged by our ability to generate higher quality poses.

### 3.1 P1: Cross‐docking extreme conformations from the naïve mapping approach

Nine‐times cross‐docking runs (as described in the Materials and Methods section) were performed using inputs generated at the extreme magnitude of the lowest normal mode (in both the positive and negative directions) for both receptor and ligand. In terms of the best FNATs achieved across the board for the 56 targets, we observe approximately a 10% uplift comparing with P0 (see Figure 1). Even more encouraging, we observe that for 21% of targets, a top docking pose can be generated that improves upon simply docking unbound receptor and ligand pairs in a single SwarmDock run (as measured by the CAPRI scoring scheme; see Table 1).

As the SwarmDock server has a number of stochastic elements associated with its central PSO algorithm, such as the generation of random initial orientations for the particles in each swarm, exactly the same docking poses are not obtained for multiple runs with identical starting protein conformations. Therefore, on 10 of the targets selected randomly, ranging from rigid body to the more difficult cases, we tested whether running SwarmDock nine times on each of them, with the standard algorithm (ie, just R(u):L(u)), would also produce improved docking poses as has been noted. Analysis of these results showed that no improved docking poses could be obtained by this simple approach (results not shown).

### 3.2 P2: Cross‐docking the theoretically closest to the bound conformations for each receptor/ligand pair

The above cross‐docking experiment indicates that better poses can be generated than simply docking the unbound. If the ideal starting conformation for each receptor and ligand could be found, ideal in as much as their conformations are closer to their bound conformation, would a SwarmDock run do as good, if not better, as the above cross‐docking protocol? To test this, the 56 docking runs were performed starting from conformations adjusted relative to the unbound by adjusting their conformations according to a linear combination of the first three lowest mode amplitudes, as shown calculated by Equation (1) the values for which are shown in Table 1. The results of these runs are shown in Figure 1. Interestingly, knowing the correct combination of normal modes, hence the generation of starting conformations closer to their bound state, did not generally improve the docking results; we observed that for only 9% of targets there was an improvement comparing with simply docking unbound receptor and ligand pairs (protocol P0), but in 14% cases the quality worsened. This may suggest that notable changes in the conformational states of both binding partners, which may even be in the opposite direction from their bound conformational states, are important in the docking process, as opposed to the simpler notion of proteins obtaining their near bound‐state conformation early and then gently descending the binding funnel with relatively minor conformational changes.

### 3.3 P3: Cross‐docking a selected energy map conformation for each receptor/ligand pair

Cross‐docking can generate docking poses with higher quality. However, the strategy employed in protocol P1 requires a 9‐fold increase in computation time relative to simply docking the unbound receptor/ligand pairs. This constitutes a considerable burden on the turnaround time for a publicly available server. Therefore, a strategy to select just one conformation for each receptor and ligand (different from simply selecting the original unbound coordinates), which only requires a 4‐fold increase in computation time, is desirable. For example, the CAPRI target (6GBG), the starting receptor structure for (R5), was created by assigning the following magnitudes (−76, 425, 240) to the minimized unbound receptor, whereas for the ligand (L5) structure (101, −64, −30) were applied.

Taking the best FNATs achieved (across the 56 targets) into consideration, we observe a similar (10%) uplift as gained by P1 (when comparing with P0; see Figure 1). We also observed that for 21% of targets, a top docking pose can be generated, which is an improvement upon simply docking unbound receptor and ligand pairs in a single SwarmDock run (as measured by the CAPRI scoring scheme; see Table 1). Considering the CAPRI methodology (which assumes that medium grade structure is also acceptable), we see an uplift in quality from both protocols P1 and P3 for 8 out of 12 complexes. Although as previously stated, the focus of this work is on generating improved docking poses as opposed to also improving their ranking, it is clearly interesting to know where these eight consistently improved poses might be ranked. Considering just the P3 protocol (4‐fold cross‐docking, equals around 2000 models), which has less final docking poses to rank than the P1 protocol (9‐fold cross‐docking, equals around 4500 models), we observed the following ranks for each of the eight targets: 1JTD ‐ 95, 3L5W ‐ 35, 4GXU ‐ 888, 3H2V ‐ 5, 1 M27‐34, 2VXT ‐ 220, 3AAD ‐ 124, and 6GBG ‐ 505. These ranks are based on merging standard clustering files produced by the SwarmDock server for each run, clusters from which are subsequently sorted by their lowest energy member.

The scoring strategy requires further innovation. However, alternative scoring schemes developed by the docking community may be far better and here we encourage scoring scheme investigations and development by making all docking poses generated in this study available for download (see Supporting Information for details on the “DYNACROSS” set available to download from https://doi.org/10.6084/m9.figshare.c.4682477).

### 3.4 General trends for each cross‐docking set

Table 1 provides the relative degree of difficulty for the 56 targets, and ranging from essentially rigid‐body docking to more difficult targets with notable conformational changes can be observed between unbound and bound components. Cross‐docking is revealed to be a valuable strategy for the entire spectrum of targets with a slightly better improvement to FNAT for rigid‐body and medium difficulty targets.

In an attempt to explain the improved conformations over P0 when compared with the strained states used in protocols P1 (maximum extension along normal mode 1) and P3 (where normalized DFIRE2 ≤ 5.0), we calculated SASA values (data not shown) for all cases with improved quality (16 in total; see Table 1). It was hypothesized that input structures are dynamic during docking and native mechanisms, such as the opening of a binding site on a receptor, which may allow easier access for incoming ligand, and would lead to an increase in SASA for the receptor. The direction of change in SASA did not prove to be a single predictor of favorable input structures, which led toward a favorable docking trajectory. This may imply that there is no obvious single feature we can use to explain why certain starting conformations are successful, as there is likely to be a complex interplay between entropic and enthalpic contributions.

### 3.5 CAPRI Target 131 (6GBG)

**Figure 2:**
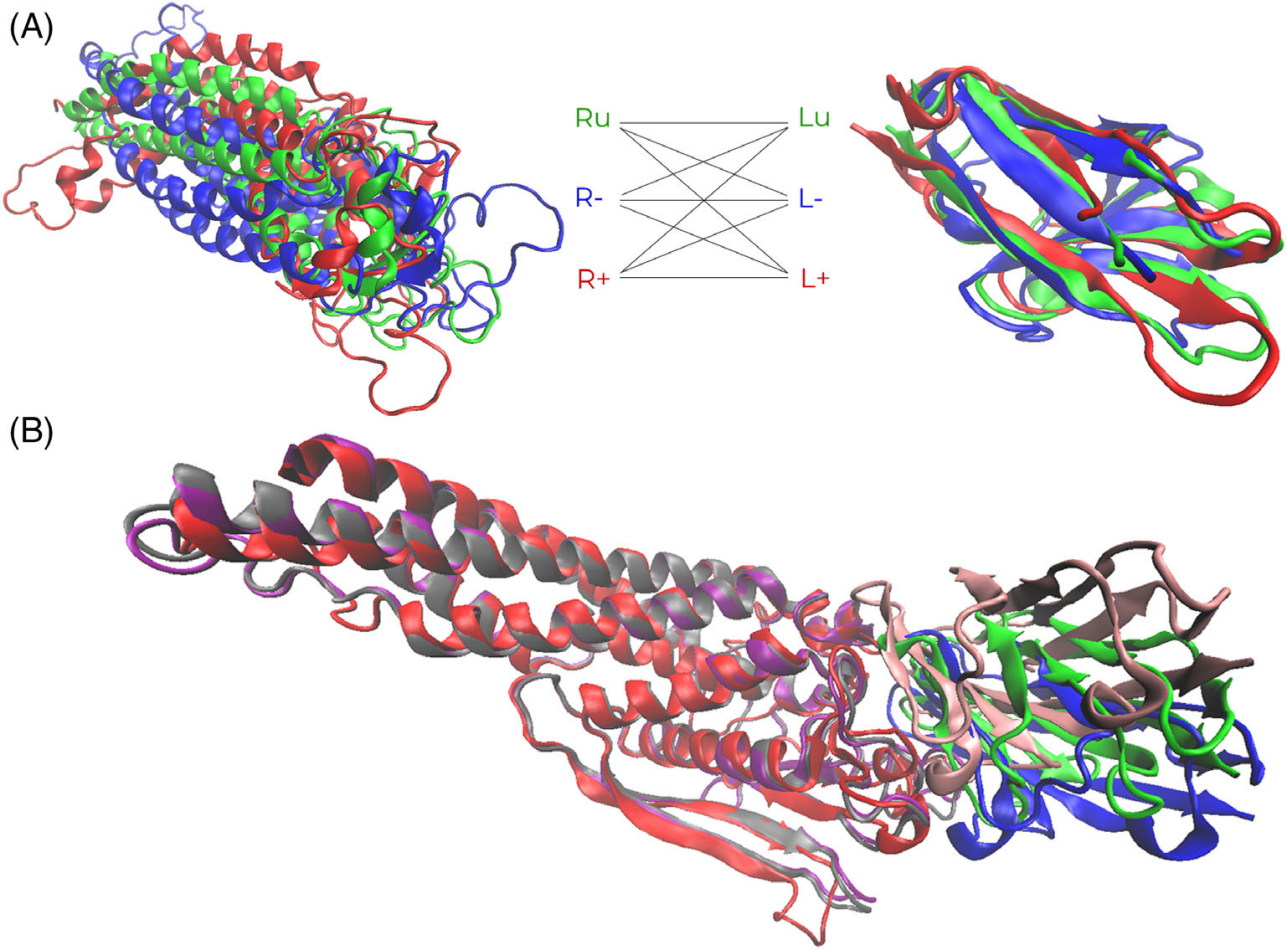
Naïve approach example—9‐fold cross‐docking conformations. A, CAPRI Target T131 (6GBG), showing the variability in conformations that are presented to the SwarmDock server. Three receptor conformations of the left, (Ru) as green, (R‐) as blue, and (R+) as red, three ligand conformations on the right, (Lu) as green, (L‐) as blue, and (L+) as red. B, The best docked pose superimposed on bound receptor structure, red and blue are for bound receptor and ligand, gray and green are for the highest quality docked receptor and ligand pair from run P1 (9‐fold docking), and purple and pink are for the highest FNAT docked receptor and ligand pair from run P0 (basic single run from unbound). The pictures were generated using VMD^26^

**Figure 3:**
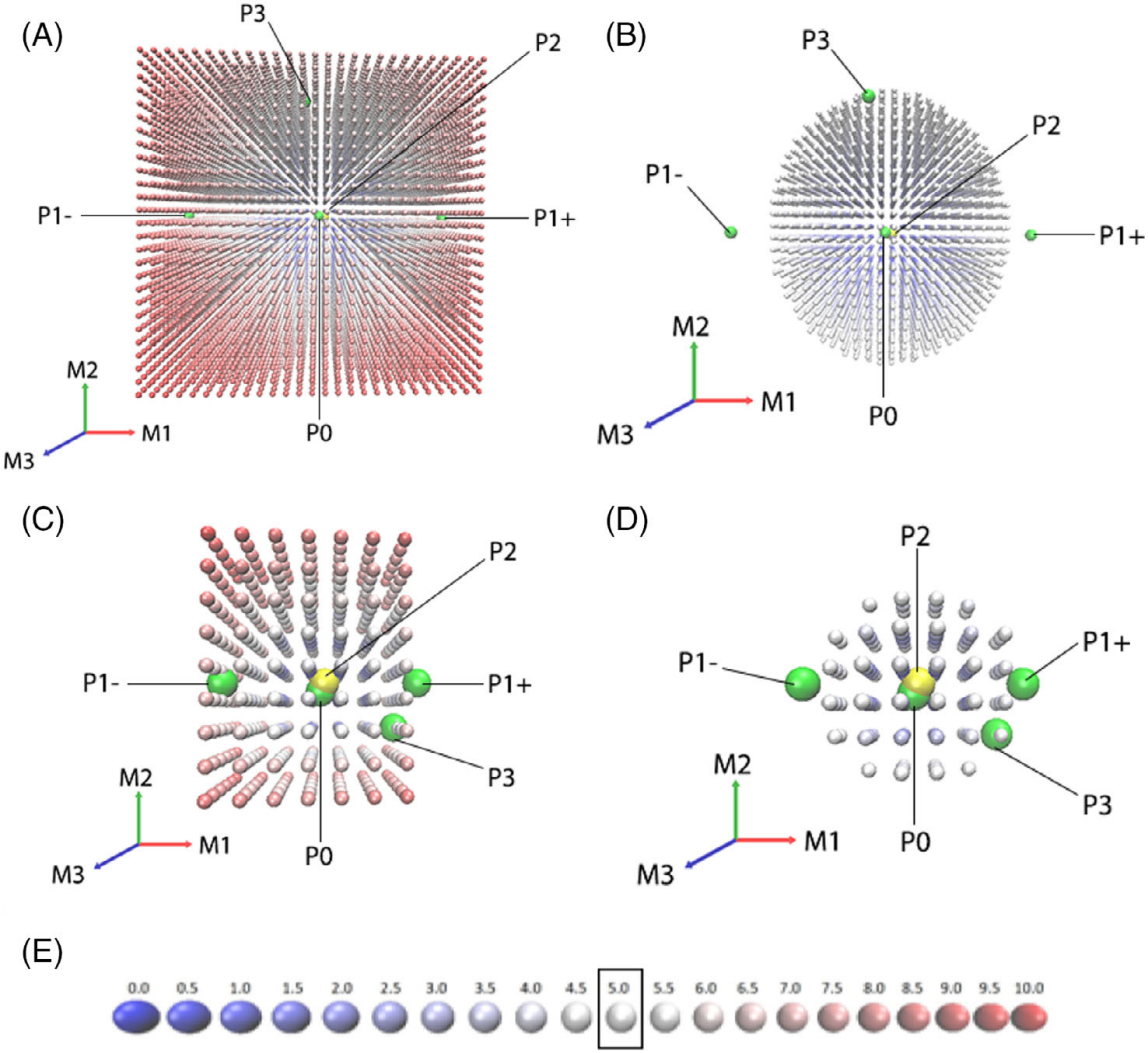
Energy diagrams for linear combinations of the first three lowest frequency modes. Receptor, all samples (A) and DFIRE2 ≤ 5.0 (B), and ligand, all samples (C) and DFIRE2 ≤ 5.0 (D) for a CAPRI Target 131, 6GBG. DFIRE2 values were normalized to the range from 0.0 (blue) to 10.0 (red) as presented in (E). Yellow marks the origin (run P0) and green marks various starting combinations as labeled in the figure for protocols P1, P2, and P3 (see the Methods section and Table 1 for details). Arrows show the normal modes axes—red, green, blue for the first, second, and third nontrivial normal mode, respectively. The pictures were generated using VMD^26^

We applied the abovementioned conformational search and cross‐docking protocol to one of the CAPRI targets (see Figure 2A): Target 131, a complex between CEACAM1, a cell surface protein receptor, and HopQ, a *Helicobacter pylori* adhesion protein.^32^ This is a target for which SwarmDock could not find an acceptable solution during the CAPRI blind trials, and the best docking pose had 0.18% of native contacts. We found acceptable solutions from (Ru):(L‐) run in protocol P1 and from (Ru):(L5) run in protocol P3 (see Figure 2B for P1 run). The conformational sampling energetic maps for receptor and ligand for the whole sampled three normal modes space and with normalized DFIRE2 ≤ 5.0 are depicted in Figure 3. It should be noted that in normal modes space, the energy distribution is not symmetrical. It must also be stated that with our final ranking scheme, we were unable to rank this improved docking pose within the top 10 runs, indicating that a parallel improvement in our ranking scheme would be needed to take full account of improvements achieved in enhancing the quality of some of our docking poses.

## 4 CONCLUSIONS

The CAPRI blind trials have set many challenges in the macromolecular docking field, ranging from docking of homology models to the construction of multibody complexes. Irrespective of the specific challenge, one clear message always feeds through in the assessment of results, and deterministic modeling of flexibility in the docking process remains an unsolved problem.^33^ One of the most exacerbating aspects, particularly for those developing docking servers, is our inability to estimate the likely degree of flexibility for each target. Counterintuitively, docking from the inputs, when the theoretically best combination of normal modes for the unbound‐bound transition is applied, is not shown to be the best docking protocol. Interestingly, the better conformations were obtained for protocols P1 and P3 where a degree of perturbation for the input structures conformations was employed. It is hypothesized that a relatively strained starting conformation could give an entropic impetus for docking. Here we show, in protocol P1, that by carefully searching the lowest frequency normal mode combinations, for both the receptor and ligand of binary complexes, conformations can be selected to enhance subsequent automated docking. Then, we assessed the energy landscape of the conformational envelope across three normal modes, up to a threshold of normalized DFIRE2 ≤ 5.0, and saw similarly positive results, but required fewer SwarmDock runs.

Essentially the method attempts to blend the two fundamental principles of associated with flexible docking—conformational selection and induced fit. The results, showing a 10% uplift in quality, compared to the simple approach of simply docking experimentally determined unbound conformations, indicate that a careful exploration of conformational selection space should ultimately aid docking efforts.

Furthermore, the previous bottleneck for sampling the conformational states space (the large number of samples and potential calculation time for each position in normal modes space) could be mitigated by applying spatial partitioning and multicore/multimachine computation. Here, we used Aether Engine, which displayed consistent speed‐ups as parallelism is added, thereby allowing one to benefit from the full amount of resources available. Therefore, the efficient preprocessing and docking pipeline could be built and run as a publicly available server, with no prior knowledge or access to supercomputing resources needed.

As an additional improvement for the future work, we consider applying Aether Engine to allow the Particle Optimization algorithm to be run on cross‐docked structures in one simulation instead of running nine (P1), or four (P3), separate SwarmDock server runs performed here. This will require the communication between swarms with various input conformations (in relation to translational and rotational space) and separately considering the normal mode space.

Future work on scoring strategies would be a good extension to this work as improving the quality of poses in the docking pipeline could only lead to improved ranking results.

As the diverse set of docking results, we named DYNACROSS for easy reference, based on varying starting conformation can be valuable for groups involved in scoring methodology development, cleaned up docking Benchmark 5 starting structures (plus CAPRI target T131) and docking results, in PDB formats, altogether with CAPRI assessment values (such as IRMSD, LRMSD, FNAT, FNONNAT, and quality), have been deposited at https://doi.org/10.6084/m9.figshare.c.4682477 (see Supporting Information for details).

## CONFLICT OF INTEREST

The authors Mieczyslaw Torchala, Patrick Gordon, Francis Russell, and Miriam Keshani of this publication (the “Publication”) are, at the time of publication, employees of Hadean Supercomputing Ltd (“the Company”). The project detailed within the Publication (the “Project”) has been entirely funded by Innovate UK, the UK's innovation agency, and the Company, at the time of publication, has no active commercial activities or plans to commercialize the Project. The terms of the arrangement between the Company and Innovate UK have been reviewed and approved by the Francis Crick Institute in accordance with its policy on objectivity in research.

## Supporting information


**Appendix** **S1**: Supplementary Material
