# Supplementary material for: Enhanced sampling of protein conformational states for dynamic cross‐docking within the protein‐protein docking server SwarmDock
Source: Proteins. 2019 Nov 20;88(8):962–72. doi: 10.1002/prot.25851 (PMC7496321; doi:10.1002/prot.25851)
Supplement: Supplementary file 1 — Appendix S1: Supplementary Material [file PROT-88-962-s001.pdf]

SUPPLEMENTARY MATERIALS

Table S1

Sampling efficiency benchmark on the selected set of proteins (R stands for receptor and L for ligand of the given complex PDB id) of various sizes without and within Aether Engine; 10 runs per every calculation were performed on the Azure cloud VM equipped with Intel(R) Xeon(R) CPU E5-2673 v4 @ 2.30GHz cores. Please see the full description in the Methods section.

Protein	Number of atoms	C++ single core implementation sampling time (seconds)	Aether Engine speed-up / cores used		
			1	2	4
4GAM_R	17445	707.6	4.37	7.70	11.56
4FQI_L	11915	599.2	3.27	5.74	8.88
4GXU_R	11382	624.7	3.13	4.79	5.99
1EXB_R	10204	645.6	2.92	4.99	7.59
3LVK_R	6117	645.6	1.47	2.41	3.39
3AAA_R	4083	609.9	1.03	1.51	1.98
3VLB_R	3012	611.6	0.57	0.85	1.14

Aether Engine

Aether Engine is a distributed simulation engine designed to bring the performance of HPC workflows to commodity clouds. It is an application of Hadean OS, which is a distributed operating system that aims to remove the need for complex programming and engineering overhead traditionally associated with HPC simulation. Hadean OS provides OS processes augmented with intrinsically distributed primitives. They optimize resource allocation and communication eliminating the need for developers to know the intricacies of distributed computing (http://hadean.com).

Aether Engine is built around a distributed octree data structure to represent 3D objects efficiently, that provisions and de-provisions compute power across an arbitrary number of cloud servers – to efficiently optimize compute whilst minimizing spend.

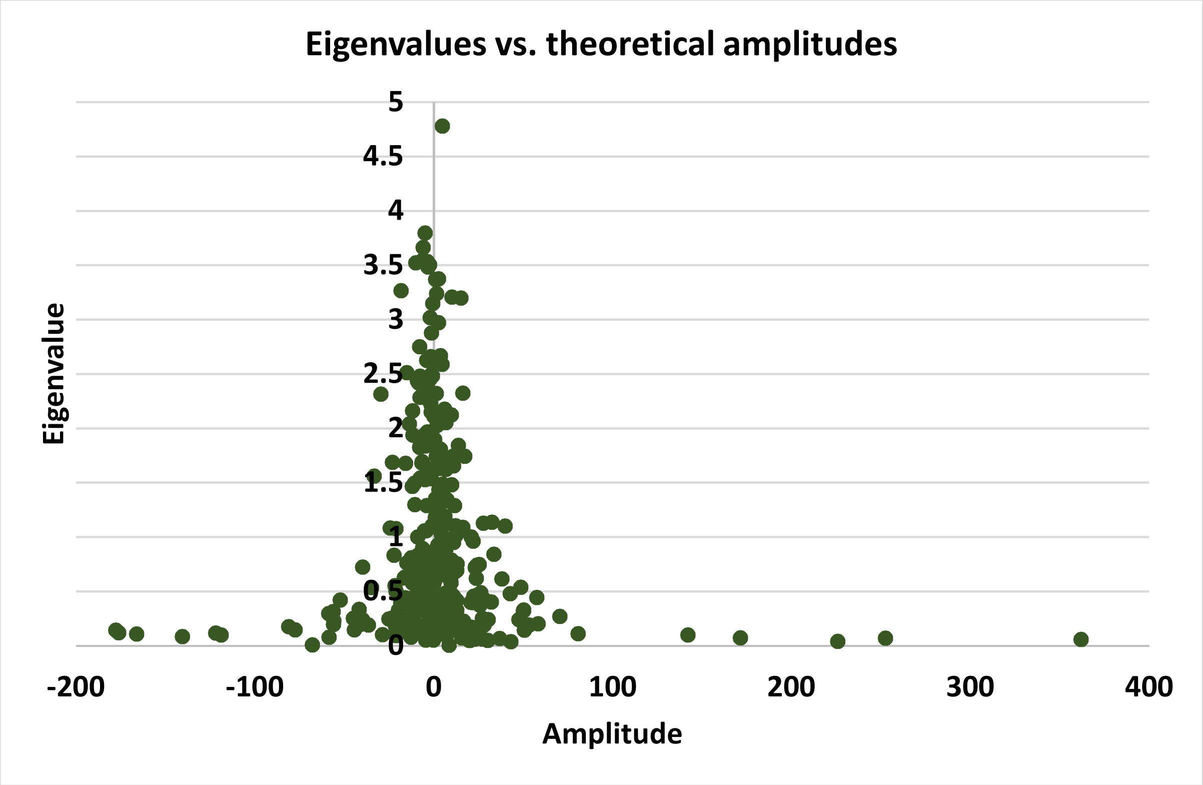

Figure S1 Eigenvalues vs. theoretical mode amplitudes for three lowest frequency modes for both receptor and ligand obtained from P2 (see Table 1 in the main text).

DYNACROSS SET (COMPILED ON 19.09.27)
https://doi.org/10.6084/m9.figshare.c.4682477

Note: all 7 files (around 29GB) need to be downloaded and unpacked together (for example under Ubuntu: 7z x DYNACROSS_190927.zip.001).

The DYNACROSS set contains two directories:

1. REPAIRED_INPUTS – this contains the repaired Benchmark 5 and CAPRI target T131 unbound and bound structures. Files denoted with 'complex' in the file name contain receptor and ligand bound structures.
2. SWARMDOCK_RESULTS – this contains compressed files returned by SwarmDock Server for all runs and text files with CAPRI assessment values (models are sorted by IRMSD in increasing order). The subdirectories for protocols are named as P1, P2 and P3.

Repair process for inputs included:

1. All missing atoms, including chain breaks, for both the unbound and bound structures, were added with the 'repair' functionality of the SwarmDock server; this process also gently minimizes the structures with a few rounds of steepest descent energy minimization.
2. All chains are numbered consistently between the unbound and bound conformations, with residues of each chain starting at number one.
3. For 3HMX receptor, heavy chain has been placed before light chain.
4. For 4GXU receptor file was swapped with ligand file to make receptor a bigger molecule, so receptor has 6 chains and ligand has 2 chains.

SwarmDock results files:

1. Full tar.gz files are deposited without any modifications as returned by the SwarmDock Server (https://bmm.crick.ac.uk/~svc-bmm-swarmdock/ webpage contains the content description).
2. The file naming convention is as follows:
   - the first four characters contains the PDB id followed by an underscore; the next four characters encode the run type;
   - set P1 contains 9 runs per complex; the run encoding part is as follows: R for receptor, L for ligand, Z for unbound, P for positive magnitude, N for negative magnitude;
   - set P2 contains 1 run per complex; the run encoding part is simply named THEO.
   - set P3 contains 4 runs per complex; the run encoding part is as follows: R for receptor, L for ligand, U for unbound, 5 for modified starting structure (the value of the normalized potential threshold); runs RULU are identical to RZLZ results already stored in P1 so they are not duplicated.
3. Please note that models coming from different runs need to be superimposed first on the common frame of reference (e.g. the bound receptor) if one wants to cluster the structures. The reason is that initial receptor and/or ligand conformations are different.

CAPRI assessment files:

1. The naming convention follows the one for the results files.
2. The assessment has been made against original Benchmark 5 bound structures in a pipeline able to find the common parts between potentially non-equivalent, in terms of the number of residues, unbound/bound structures.
3. For every model IRMSD, LRMSD, FNAT, FNONAT and quality are reported. The models are sorted by IRMSD in the increasing order.
4. Some bigger complexes contain the equivalent chains, so they were assessed in all possible cyclic shifts of the equivalent chains (the last chain becoming the first chain). Therefore, for some complexes, there are additional text files with _SHIFTS_ substring in the file name, additional permutations for:
    - receptor files for 3P57, BP57, 3R9A, 4JCV (3 shifts), 4LW4, CP57, 3L89 (2 shifts), 4GAM, 4GXU (2 shifts), 3LVK, 4HX3;
    - ligand files for 3EO1 and 4FQI (2 shifts).
5. RZLZ run in P1 is equivalent to RULU run in P3 so assessment files are not duplicated.
